# Nerve-specific extracellular matrix hydrogel promotes functional regeneration following nerve gap injury

**DOI:** 10.1038/s41536-021-00174-8

**Published:** 2021-10-25

**Authors:** T. Meder, T. Prest, C. Skillen, L. Marchal, V. T. Yupanqui, L. Soletti, P. Gardner, J. Cheetham, B. N. Brown

**Affiliations:** 1 McGowan Institute for Regenerative Medicine, University of Pittsburgh, Pittsburgh, PA USA; 2 Department of Bioengineering, Swanson School of Engineering, University of Pittsburgh, Pittsburgh, PA USA; 3 Renerva, LLC, Pittsburgh, PA USA; 4 Department of Neurological Surgery, University of Pittsburg School of Medicine, University of Pittsburgh, Pittsburgh, PA USA; 5 Department of Clinical Sciences, Cornell College of Veterinary Medicine, Cornell University, Ithaca, NY USA

**Keywords:** Translational research, Biomedical materials, Somatic system, Neurophysiology

## Abstract

Nerve transection requires surgical intervention to restore function. The standard of care involves coaptation when a tension-free repair is achievable, or interposition of a graft or conduit when a gap remains. Despite advances, nerve gap injury is associated with unsatisfactory recovery. This study investigates the use of a decellularized, porcine nerve-derived hydrogel filler (peripheral nerve matrix, PNM) for conduits in an 8 mm rat sciatic nerve gap model. The decellularized tissue maintained multiple nerve-specific matrix components and nerve growth factors. This decellularized tissue was used to formulate hydrogels, which were deployed into conduits for nerve gap repair. Nerve recovery was assessed up to 24 weeks post injury by gait analysis, electrophysiology, and axon counting. Deployment of PNM within conduits was shown to improve electrophysiologic response and axon counts compared with those of empty conduit controls. These results indicate that PNM has potential benefits when used as a filler for conduits in nerve gap injuries.

## Introduction

Acute peripheral nerve injuries (PNI) are observed in 3.85% of all trauma admissions^1^ as well as in up to 0.11% of all surgical procedures as a result of iatrogenic injury^2,3^. Together, these two sources alone account for >200,000 cases in the United States annually. PNI can result in permanent functional deficits with severe impacts on the quality of life and productivity of those affected^4–7^. The degree to which PNI patients experience neuropathic pain, sensory and motor deficits, and other sequalae is dependent on injury location, type, and severity. These same variables inform the technique used for surgical repair as well as the prognosis for functional recovery. Mild-to-moderate compressive or stretch injuries can recover without surgical intervention, whereas more severe injuries such as transection must be surgically repaired for meaningful recovery to occur^8^. When achievable, transection injuries are treated with simple coaptation of the nerve ends; however, when a gap remains, nerve autografts, allografts, or conduits are commonly used for the repair. Allografts and conduits have the advantage of repair without the donor site morbidity and functional losses associated with the use of autografts^8,9^. However, allografts are only considered effective for gap lengths smaller than 5 cm^10^, nerve conduits are only considered effective for gap lengths smaller than 3 cm^10,11^, and neither allograft nor conduit repairs routinely achieve the functional results seen with autografts. Thus, there is a need for solutions that improve the outcome of nerve gap repair without resorting to autograft harvest.

Preclinical studies in both small and large animal models have shown that nerve conduit performance can be enhanced through the use of luminal fillers with structures that are aligned along the long axis of the conduit^12–18^, sustained release of neurotrophic factors^19–25^, or through the addition of Schwann and/or stem cells^26–31^. However, substrates within the lumen have also been shown to hinder healing if the filler is not sufficiently amenable to Schwann cell (SC) migration and/or axonal penetration and elongation^32–34^. This is particularly true for dense, unaligned collagen substrates and is similar to the effects of fibrous scar tissue deposition^14,16,32,33^. It has also been observed in vitro that SCs and neurites prefer to travel towards softer stiffness substrates^35,36^, suggesting that materials within the lumen which are stiffer than the surrounding tissues can be inhibitory to nerve growth. These limitations, along with technical and regulatory hurdles associated with the use of neurotrophic factors and stem cells, have likely prevented the translation of luminal fillers for nerve conduits into clinical practice.

**Fig. 1:**
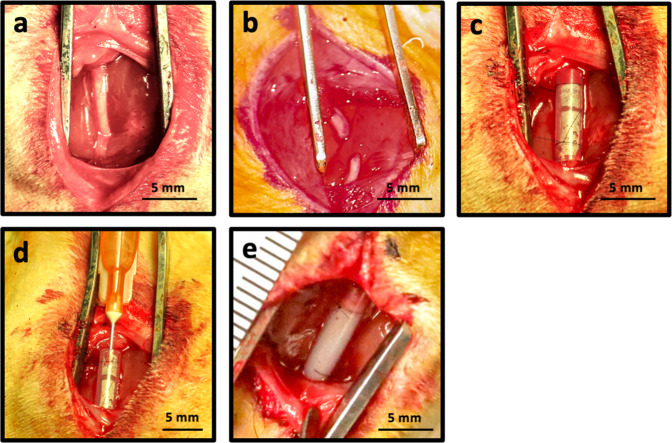
Subcritical gap injury model and application of PNM. **a** The left sciatic nerve is exposed at least 5-mm proximal to the sciatic bifurcation. **b** The nerve is transected, and an 8 mm segment is resected. **c** Nerve stumps are sutured 1 mm into each end of a 10 mm silicone conduit (2 mm inner diameter). **d** PNM is delivered into the conduit. **e** Gelation occurs within 5 minutes following delivery.

This study describes the derivation and characterization of a decellularized, porcine nerve-derived, extracellular matrix hydrogel (peripheral nerve matrix, PNM), and examines its use as a luminal filler for silicone nerve conduits in a rodent nerve gap defect model (Fig. 1). Decellularized tissues were examined for nerve-specific extracellular matrix and growth factor composition, and the resultant hydrogels were characterized at the mechanical and structural levels. Subcritical nerve gap defects (8 mm) were then created in the sciatic nerve of Fisher rats and treated using PNM-filled conduits. Recovery was assessed over a 24-week period using longitudinal analysis of limb function, end study electrophysiologic testing, and axon counting in histologic sections. Recovery associated with PNM-filled conduits was compared to that of empty conduits and autograft controls. An additional study was then performed to determine the impact of PNM concentration upon outcomes.

## Results

### Characterization of decellularized peripheral nerve

**Fig. 2:**
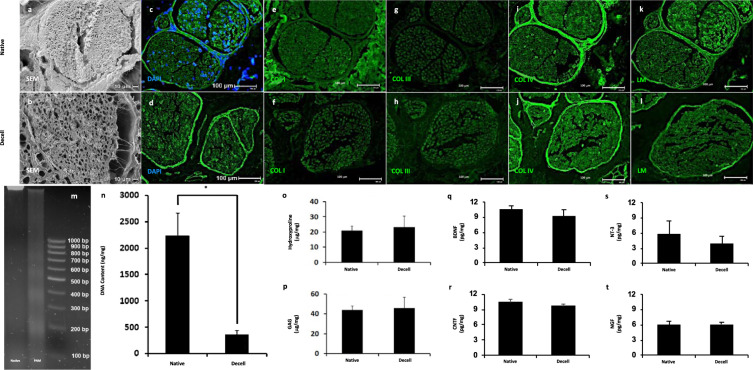
Characterization of decellularized porcine peripheral nerve. SEM imaging before **a** and after **b** decellularization showed that the structure of the nerve was maintained following the decellularization process. Scale bars 10 µm. DAPI labeling of native **c** and decellularized **d** tissue showed the removal of nuclei in the decellularized samples. Additional labeling for collagen I (**e**, **f**), III (**g**, **h**), IV (**i**, **j**), and laminin (**k**, **l**) showed strong maintenance of key nerve proteins collagen IV and laminin in the decellularized samples. Scale bars 100 µm. An agarose gel was used to assess the fragmentation of any remaining DNA within the sample (**m**) and a PicoGreen assay (**n**) was used to demonstrate a significant (*p < 0.001) reduction in dsDNA content. Biochemical assays were used to demonstrate the maintenance of hydroxyproline (**o**) and GAG (**p**) content. ELISA assays were used to assess the maintenance of nerve growth factors BDNF (**q**), CNTF (**r**), NT-3 (**s**), and NGF (**t**). Data are presented as mean ± SD. All blots were derived from the same experiment and processed in parallel.

Decellularization resulted in the maintenance of nerve architecture and composition to a high degree. SEM showed dense tissue between and within individual fascicles before decellularization (Fig. 2a). After decellularization, the tissue was observed to be porous with empty tubules where axons previously resided (Fig. 2b). The original structures, including endoneurium and perineurium were preserved. The further assessment confirmed that the tissue was effectively decellularized (Fig. 2c, d) and that the structure and composition of the tissue were also largely maintained. Native and decellularized nerve exhibited positive labeling for collagen I (Fig. 2e, f), collagen III (Fig. 2g, h), collagen IV (Fig. 2i, j), and laminin (Fig. 2k, l). Collagen I, seen predominately within epineurial spaces around the nerve fascicles, was reduced post-decellularization. Limited positive staining of collagen III was seen in native and decellularized nerves. Collagen IV and laminin were found to be largely preserved.

Gel electrophoresis demonstrated that the remaining DNA was highly fragmented (Fig. 2m), and a PicoGreen assay for dsDNA showed an 84% (*p* < 0.01) reduction in residual dsDNA after decellularization (Fig. 2n). No significant differences were observed in either hydroxyproline (Fig. 2o) or glycosaminoglycan (Fig. 2p) content. Assessment of nerve-specific growth factors (BDNF, Fig. 2q; CNTF, Fig. 2r; neurotrophin-3 (NT-3), Fig. 2s; nerve growth factor (NGF); Fig. 2t) showed no significant differences between native and decellularized tissues.

### Characterization of nerve-specific hydrogel

**Fig. 3:**
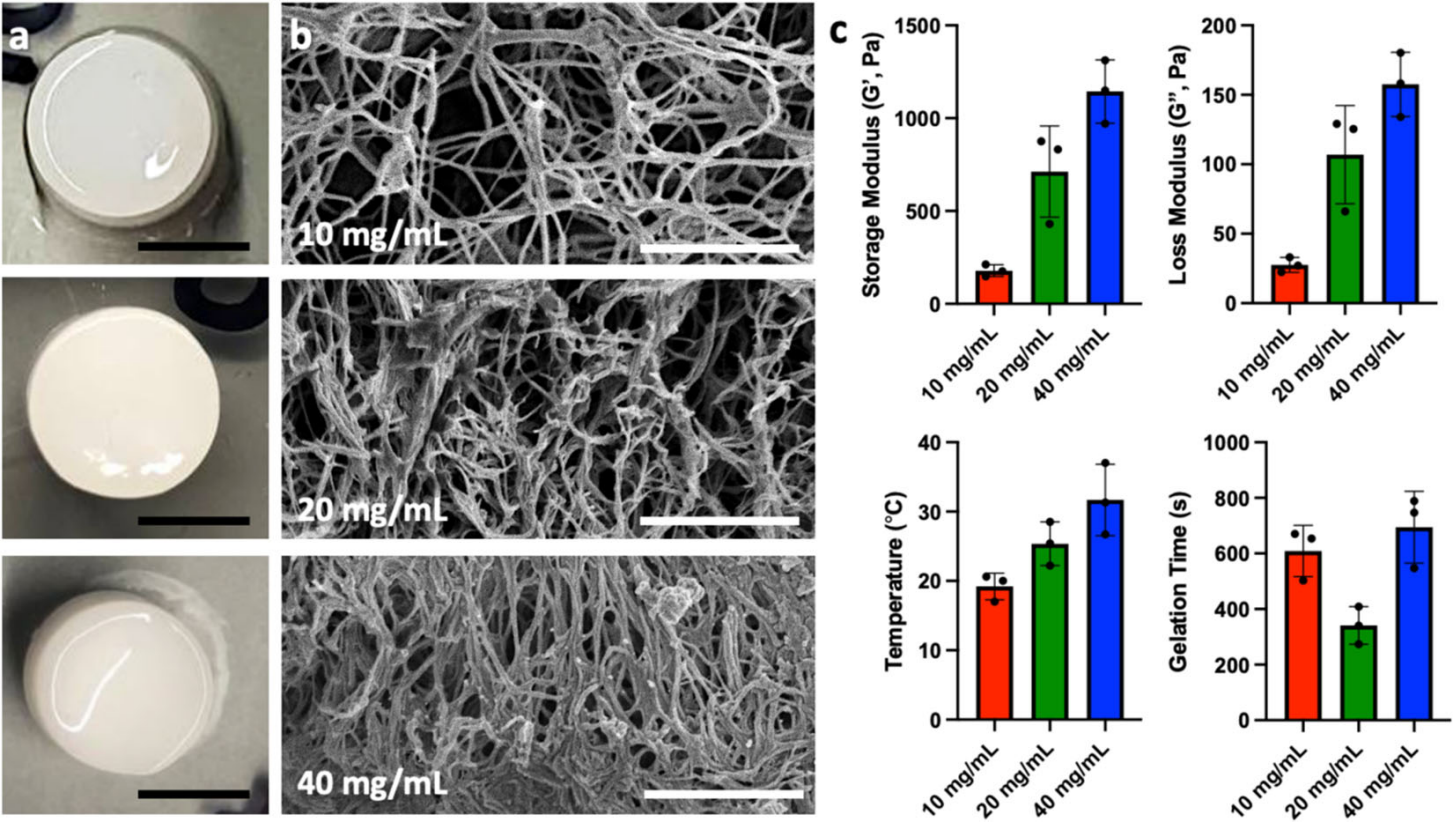
Characterization of decellularized nerve tissue-specific hydrogel. Macroscopically, hydrogels were formed at all protein concentrations, but the ability of the material to retain fluid was observed to increase at higher concentrations (**a**). Scale bars 0.5 cm SEM imaging was used to examine the microstructure of gels resulting from protein concentrations of 10 mg/mL, 20 mg/mL and 40 mg/mL (**b**). Increasing fiber density was observed with increased protein concentration. Scale bars 2.5 μm. Representative curves from rheologic testing, varying protein concentration (**c**) are shown. Storage modulus, loss modulus, gelation temperature, and gelation time are shown (**c**). Data are presented as mean ± SD.

Robust hydrogels were formed at concentrations of 10, 20, and 40 mg/mL (Fig. 3a). At the macroscopic level, 10 and 20 mg/mL gels retained more liquid than the 10 mg/mL concentrations. Structurally, the gels were characterized by a highly porous network of collagen bundles (Fig. 3b). Increased fiber density was observed as the protein concentration of the gel increased. The effects of gel concentration upon storage modulus, loss modulus, gelation temperature, and gelation time were evaluated (Fig. 3c). Increases in storage modulus (178.7 ± 32.0, 712.3 ± 245.3, 1144.0 ± 170.3 Pa) and loss modulus (27.5 ± 5.3, 106.9 ± 35.3, 157.5 ± 23.1 Pa) were observed with increasing gel concentration. Gelation temperature was also found to increase with increasing gel concentration (19.2 ± 1.9, 25.4 ± 3.2, 31.7 ± 5.2 °C). Gelation time was found to be shortest in the 20 mg/mL concentration (3441 ± 68 s) compared with the 10 and 40 mg/mL concentrations (609 ± 92, 694 ± 129 s). PNM at each concentration was easily delivered through a 25-gauge needle, was observed to gel within the silicone conduit, and remained within the conduit during surgical closure (Fig. 1).

### Longitudinal gait analysis

**Fig. 4:**
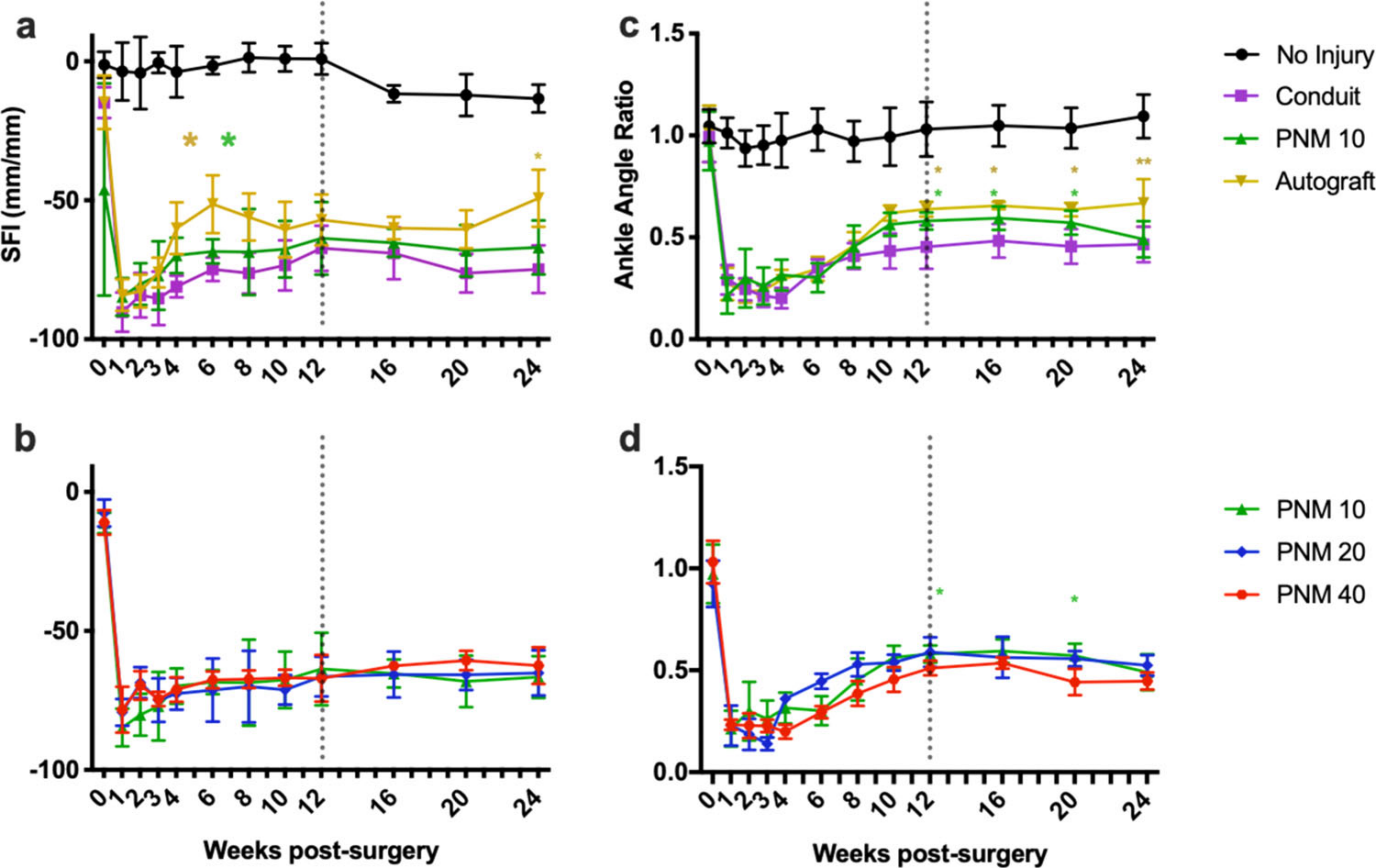
Gait analysis via SFI and ankle angle measurements in experimental and NI groups normalized by their contralateral hind limb values. Vertical dotted line placed at the threshold for the piecewise statistical approach described in the methods section. **a** SFI over 24 weeks. Gold asterisks denote significance between autograft and PNM-10 and green asterisks denote significance between PNM-10 and conduit. **b** Ankle angle over 24 weeks. **c** SFI over 24 weeks for PNM groups. Gold asterisks represent significance when comparing autograft to PNM-10 and green asterisks represent significance when comparing PNM-10 to the conduit. **d** Ankle angle over 24 weeks for PNM groups. A green asterisk compares PNM-10 with PNM-40. *P* values <0.05 are denoted by *, *p* < 0.01 by **, *p* < 0.001 by ***, and *p* < 0.0001 by ****.

Immediately following nerve injury, both SFI and ankle angle decreased sharply in all experimental groups. A regenerative response is observed following nerve injury until ~10–12 weeks when values begin to plateau until 24 weeks. Over the first 12 weeks, significant differences were observed overall in the linear mixed model comparison (*p* < 0.001 for treatment) (Fig. 4a). Individual comparisons demonstrated a greater rate of functional recovery in the autograft group as compared with PNM-10 (+6.257 mean difference, *p* = 0.009) and a greater rate of functional recovery in PNM-10 group as compared with the conduit group (+6.549 mean difference, *p* = 0.009). Time was found to have a significant impact (*p* < 0.001), indicating significant functional gains in all groups over time. After 12 weeks, no differences were observed between PNM-10 and Autograft or PNM-10 and Conduit until week 24 where Autograft showed significant improvements over PNM-10 (26.4% increase, −49.29 ± 10.28 vs. −66.96 ± 9.71, *p* = 0.0409).

No differences were observed over the first 12 weeks when comparing ankle angle ratio (*p* = 0.783 for treatment), however significant functional gains did occur over time in all groups (*p* < 0.001 for time) (Fig. 4c). No differences were observed in the rate of increase in the ankle angle when comparing PNM-10 with Autograft (*p* = 0.884) or PNM-10 to Conduit (*p* = 0.603). Comparisons at weeks 12, 16, 20, and 24 revealed significant differences in extent of recovery, with PNM demonstrating superiority to Conduit (28.8%, 18.6%, and 20.3% greater; 0.580 ± 0.042 vs. 0.452 ± 0.107, 0.594 ± 0.057 vs. 0.483 ± 0.082, 0.571 ± 0.058 vs. 0.455 ± 0.085; *p* = 0.0216, 0.0155, 0.0143; at weeks 12, 16, and 20, respectively). However, Autograft showed significantly higher values than PNM (9.1%, 9.4%, 10.2%, and 26.4% greater; 0.638 ± 0.036 vs. 0.580 ± 0.042, 0.656 ± 0.018 vs. 0.594 ± 0.057, 0.636 ± 0.033 vs. 0.571 ± 0.058, 0.668 ± 0.117 vs. 0.491 ± 0.088; *p* = 0.0195, 0.0342, 0.0369, 0.0083 at weeks 12, 16, 20, and 24, respectively).

When comparing PNM concentration, no differences were observed within the first 12 weeks (*p* = 0.339 for SFI and *p* > 0.999 for ankle angle) (Fig. 4c, d). All concentrations performed equivalently for SFI and ankle angle when comparing the rate of recovery, but some differences were seen when observing the extent of recovery. At weeks 12 and 20, ankle angle was significantly decreased in PNM-40 compared to PNM-10 (11.8% and 22.6% difference, *p* = 0.0449, 0.0380 at 12 and 20 weeks, respectively).

### Electrophysiological assessment

**Fig. 5:**
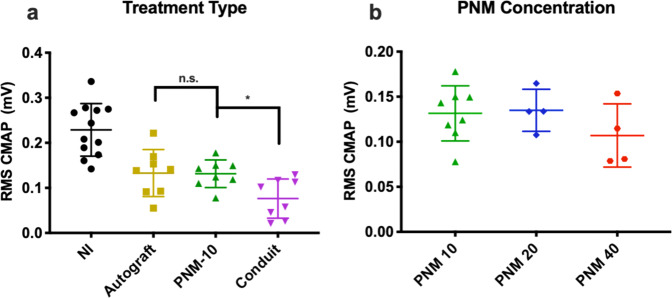
Electrophysiological assessment was performed immediately prior to sacrifice at 24 weeks. **a** CMAP at 24 weeks comparing the treatment types. **b** CMAP at 24 weeks comparing the PNM groups. Data are presented as mean ± SD. *P* values <0.05 are denoted by *, *p* < 0.01 by **, *p* < 0.001 by ***, and *p* < 0.0001 by ****.

Results of electrophysiological assessment at 24 weeks are shown in Fig. 5. The use of PNM-10 as a luminal filler was associated with improved compound motor action potential (CMAP) as compared with the conduit-only group (0.131 ± 0.031 vs. 0.076 ± 0.044 mV, *p* = 0.043; Fig. 5a). The PNM-10 group was found to have CMAP that was statistically equivalent to the autograft group (0.133 ± 0.052 mV and 0.131 ± 0.031 mV respectively, *p* > 0.999). No significant differences in CMAP were detected between different concentrations of PNM (PNM-10, 0.131 ± 0.031 mV; PNM-20, 0.135 ± 0.023 mV; PNM-40, 0.107 ± 0.035 mV, *p* > 0.972 for all comparisons), though values were lowest for the PNM-40 group.

### Axon counting

**Fig. 6:**
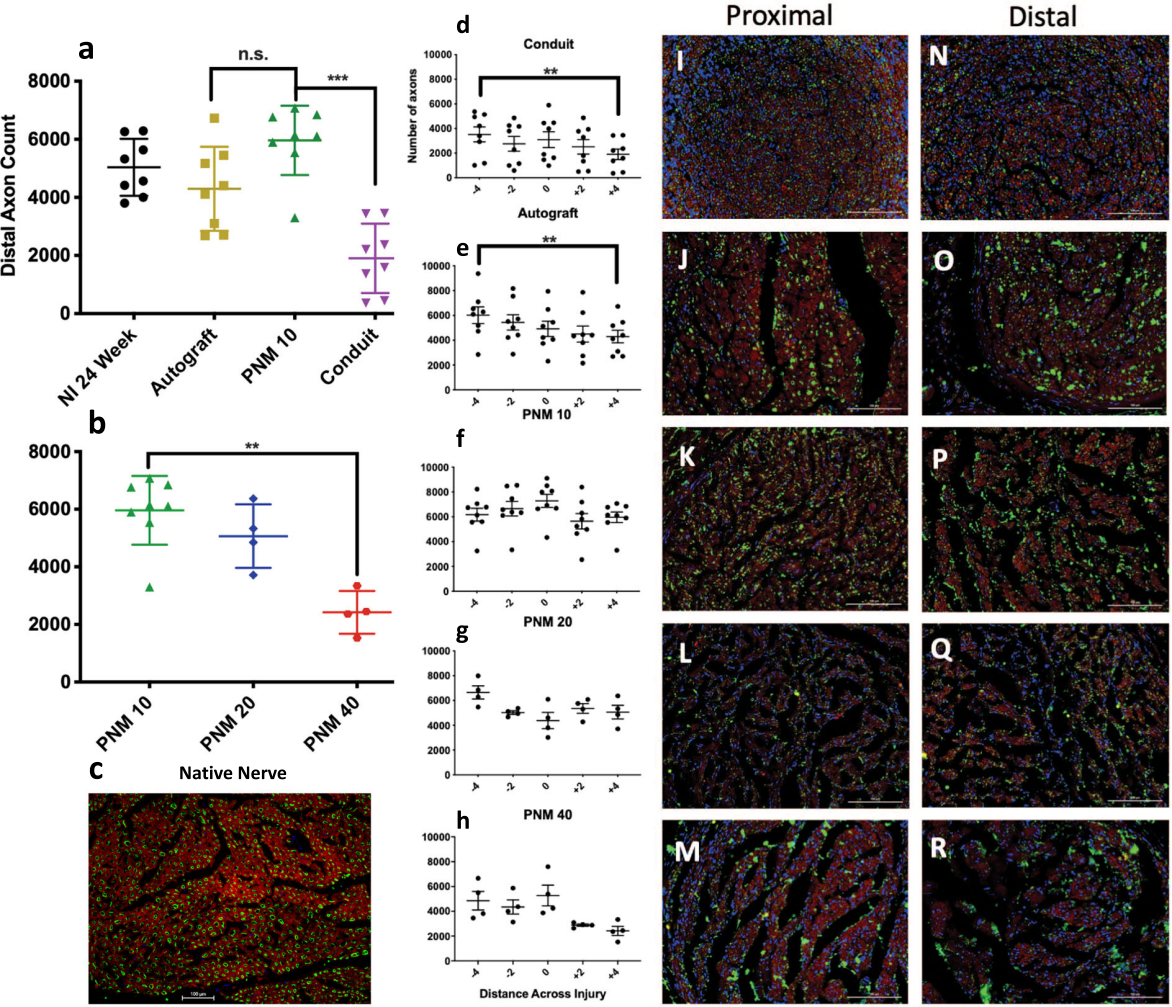
Axon quantification and qualitative morphological appearance. **a** Axon counts for each group. **b** Comparisons of axon counts between concentrations of PNM. **C** Representative cross-section of a native, non-injured rat sciatic nerve at ×20 magnification. Scale bar 100 µm, neurofilament heavy chain for axons tagged in green, fluoromyelin for myelin in red, and DAPI for nuclei in blue. **d**–**h** Longitudinal distributions of axon count for each group. **i**–**m** Representative sections at the proximal (−4 mm) and **n**–**r** distal (+4 mm) locations of the gap defect. The scale bar represents 100 μm and images were taken at ×20 magnification. Axons appear in green, myelin in red, and nuclei in blue. Data are presented as mean ± SD. *P* values <0.05 are denoted by *, *p* < 0.01 by **, *p* < 0.001 by ***, and *p* < 0.0001 by ****.

Results of the histological assessment of axon number at proximal and distal ends of the repair site are shown in Fig. 6. The most distal sections were used to compare axon quantity between groups (Fig. 6a, b). Within-group comparisons were also made between proximal and distal ends of the repair site (Fig. 4d–h). PNM-10 showed a statistically significant increase in axon number at the distal end as compared to the conduit-only group (5962 ± 1193 vs 1903 ± 1194, *p* < 0.001; Fig. 6a). No statistical differences were observed between the PNM-10 and autograft groups (5962 ± 1193 vs. 4295 ± 1446 axons, *p* = 0.154). No statistical differences were observed between PNM-10 and PNM-20 groups (5962 ± 1193 vs. 5066 ± 1101 axons, *p* = 0.972; Fig. 6b) but PNM-40 was found to have significantly fewer axons than the PNM-10 group (2421 ± 741 axons vs. 5962 ± 1193, *p* = 0.009; Fig. 6b). Additional analyses were performed to assess decreases in axon number between proximal and distal ends. Significant decreases in axon number were found at the distal end for autograft (6108 ± 1893 vs. 4295 ± 1446 axons, *p* = 0.008; Fig. 6e) and conduit groups (3523 ± 1719 vs. 1904 ± 1194 axons, *p* = 0.008; Fig. 6d) No significant differences were found for PNM-10 (6170 ± 1460 vs. 5962 ± 1193 axons, *p* = 0.9453; Fig. 6f), PNM-20 (6649 ± 1058 vs. 5066 ± 1101 axons, *p* = 0.125; Fig. 6g), or PNM-40 (4857 ± 1497 vs. 2421 ± 741 axons, *p* = 0.125; Fig. 6h) groups. Representative images of the native nerve (Fig. 6c) and nerve repair groups at proximal and distal locations are shown in Fig. 6i–q.

## Discussion

The present study examines the efficacy of a decellularized, porcine PNM-derived hydrogel used as a luminal filler for silicone nerve conduits in subcritical nerve gap defects in rats. Decellularized tissues were observed to retain a significant degree of nerve-specific extracellular matrix components as well as nerve growth factors. The subsequent hydrogels were assessed for structure and mechanical moduli, with the density and modulus of the material increases with increased concentration. PNM-10 was chosen as the main experimental group as increasing lumen filler density and stiffness have been associated with decreased cellular migration and nerve regeneration^14,16,32,33,35,36^. Nerve healing in an 8 mm gap defect model was assessed via gait analysis, electrophysiology, and histology. These metrics suggested that, in this injury model, gap repair with conduits filled with PNM-10 outperformed empty conduit controls, and approached recovery observed in the autograft group in many metrics. Increasing PNM concentration beyond PNM-10 suggested progressively decreased efficacy in promoting nerve regeneration, though differences were only observed between the PNM-10 and PNM-40 groups.

Previous animal studies of conduits filled with multiple substrates, bioactive factors, and cellular constituents have demonstrated improvements in outcome. Luminal fillers improving SC migration by providing longitudinally oriented substrates or by releasing neurotrophic factors have been particularly effective at promoting nerve regeneration^14,15,19,26^. However, luminal fillers made of dense substrates with unaligned fibrous structures, such as those made of extracellular matrix including collagen or fibrin, have demonstrated fewer axons and overall poorer functional outcomes compared with their respective empty controls^14,16,32,33^. This delicate combination is one potential explanation for the decreased efficacy seen with increasing PNM concentration in the present study. That is, higher ECM concentrations may inhibit cellular migration owing to the stiffness or density of the material within the conduit.

PNM consists exclusively of the enzymatically digested components of the decellularized porcine sciatic nerve. No exogenous bioactive factors or cells are added to the material, and no modifications are made to align ECM components within the hydrogel, which has an unaligned and interwoven fibrous structure, as observed under SEM. Despite the lack of a longitudinally aligned structure, a previous study demonstrated that PNM is an effective scaffold for SC migration^37^. Additional studies have also demonstrated that PNM promotes a shift in macrophage polarization from a predominantly M1, pro-inflammatory phenotype to an M2-like, anti-inflammatory, and pro-healing phenotype, which has been associated with improvements in peripheral nerve repair^38–40^. Thus, this and previous studies^41^ show that PNM has nerve regenerative potential when used as a scaffold for nerve regeneration, despite lacking fiber alignment or the addition of exogenous factors or cells. This is a feature that is not commonly observed in other studies describing luminal fillers, and particularly those using extracellular matrix-based components. The exact mechanisms by which PNM hydrogels promote shifts in the inflammatory response and promote downstream function improvements remain unknown and are the subject of ongoing work.

In the present study, a subcritical 8 mm defect was created in the rat sciatic nerve. Although below the critical defect size threshold in the rat (1.5 mm)^42^, 8 mm is within the viable range for conduit application and is representative of clinical scenarios in which the surgeon would elect to perform either a direct coaptation and neurorrhaphy or choose a bridging option between autograft, allograft, or conduit^43^. In the present study, gap defects were repaired immediately following the creation of the gap defect using a non-porous and inert silicone conduit with and without PNM as a luminal filler. Silicone conduits, though not frequently used in clinical practice, are inert and allowed for a more isolated assessment of the efficacy of PNM as a luminal filler. The gap length of 8 mm was also informed by this choice, as non-porous conduits have been observed to fail in gaps >10 mm owing to a lack of vascular support and nutrient/waste exchange^32^. Future studies will examine the application of PNM within clinically relevant, degradable conduits and with longer gap lengths.

Gait analysis over 24 weeks in all treatment groups demonstrated an initial sharp decrease in SFI and ankle mobility following gap injury, as expected, followed by 10–12 weeks of progressively increased functional recovery prior to reaching a plateau. Greater rates of recovery for the PNM group compared with the empty conduit group were observed; however, no differences in terminal recovery were observed. No improvements in the rate of recovery of ankle angle were found; however, improvements in end terminal function were observed between the PNM and empty conduit groups. This suggests multiple improvements in functional recovery associated with the use of PNM. However, gait compensatory effects are known to cause an increased variance, which is a commonly observed phenomenon when measuring gait in rodents^44^. In addition, it is believed that the permanent presence of a bio-inert silicone conduit can impair later nerve healing after the regenerative bridge has been established^45^. Therefore, is possible that greater impact could be achieved with the use of a biodegradable conduit as opposed to the silicone conduit employed in this study and future studies could employ more sensitive functional outcome metrics such as force generation and should address potential improvements in reinnervation at the muscle level through the analysis of neuromuscular junction formation.

Nerve electrophysiology and axon quantification demonstrated more clearly defined differences among treatment groups. In particular, PNM-10 was found to be superior to the empty conduit group and matched the performance observed with autografts. Assessments of differences in axon number between proximal and distal ends of the repair site showed significant decreases for both the autograft and conduit groups. This suggests that growth was slowed or inhibited along the length of the repair. The present study did not perform histologic assessments of material degradation and remodeling over time, and future studies are needed to assess the impact of the early remodeling process on axonal growth and functional recovery over time.

Although PNM at 10 mg/mL and 20 mg/mL showed similar regenerative benefits, PNM at 40 mg/mL concentrations displayed a diminished regenerative capacity as compared with the 10 mg/mL group. Combined with observations of increasing density and substrate modulus, this suggests an impairment of axon penetration through the luminal filler when the concentration of PNM is increased. Although the exact reasons are not known, we hypothesize that this could be an effect of the increased density and stiffness of the material, both of which have been shown in previous studies to have potentially detrimental impacts upon cellular migration and axonal elongation across nerve gaps^32,33,35–37^. The present study did not establish the minimum or maximum concentrations which can be achieved with the PNM hydrogel, and future studies are needed to assess whether additional gains in functional recovery could be achieved as well as to directly compare PNM to other candidate hydrogel substrates.

PNM as a luminal filler for inert silicone conduit was shown to produce gait, electrophysiologic, and histologic outcomes equivalent to autografting for subcritical nerve gap injuries over or at 24 weeks in a rat model. PNM demonstrated superior performance in comparison with control conduits filled with saline; however, an upper limit on effective PNM concentration was observed. Future work is needed to assess muscle reinnervation and to demonstrate the effectiveness of PNM in critical gap defect models as well as more proximal injury models in larger animals. In addition, a combination of PNM with porous degradable conduits may enhance regenerative capacity as compared to the silicone conduits used in the present study. However, the results of the present study suggest that PNM-10 is a promising luminal filler for nerve conduits to enhance nerve regeneration in nerve gap injury.

## Methods

### Preparation of nerve-specific hydrogels

Sciatic nerves were collected from market-weight pigs and stored at −80 °C until use. Tissues were extracted from animals post-mortem, no procedures were performed on live pigs in the collection process. The tissue was thawed in Type I water at room temperature, and excess connective tissue was manually removed. The tissue was then quartered longitudinally and cut into sections of <5 cm. These tissue sections were soaked for 15 minutes in Type I water without agitation and excess lipid was decanted from the top liquid layer; this process was repeated until all visible lipid was removed. A series of agitated washes were then performed including Type I water for 14 hours at 4 °C, 0.02% trypsin/0.05% EDTA solution for 60 mins at 37 °C, 3.0% Triton X-100 for 60 minutes at room temperature, 1 M sucrose for 15 mins at room temperature, 4.0% sodium deoxycholate for 60 mins at room temperature, and 0.1% peracetic acid/4% ethanol for 120 mins at room temperature to disinfect and depyrogenate the tissues. Tissues were again washed in 1× PBS for 15 minutes at room temperature followed by 14 hours in type I water at room temperature, and one final 1× PBS wash for 15 mins at room temperature to remove residual detergents or enzymatic agents and return the tissue to physiologic pH. Next, the tissue was frozen at −80 °C and then lyophilized for 48 h.

To digest the lyophilized, decellularized nerve, tissue was diced and solubilized at a concentration of 20 mg of ECM/mL in a solution containing 1.0 mg/mL pepsin in 0.01 M HCl at a constant stir rate of 1400 RPM for 48 hours at room temperature. The resulting digested ECM solution was then aliquoted, frozen at −80 °C, and subsequently lyophilized for 48 hours. Immediately prior to application, the lyophilized digest was reconstituted and neutralized on ice to 7.4 pH at an ECM concentration of 10, 20, and 40 mg of ECM/mL. Neutralization was achieved by adjusting the pH of the reconstituted digest to 7.4 through the addition of one-ninth digest volume of 10× PBS followed by 0.2 M NaOH until reaching 7.4 pH, and then diluting to the desired final ECM concentration in 0.5× PBS. This process was originally described in a prior publication^41^.

### Characterization of nerve-specific hydrogels

The impact of processing upon the maintenance of tissue-specific extracellular matrix and growth factor components within decellularized nerve tissue was assessed by scanning electron microscopy, immunolabeling, biochemical assays, and enzyme-linked immunosorbent assay (ELISA). A portion of the decellularized nerve was fixed in glutaraldehyde, processed through an increasing series of ethanol washes, and critical point dried for observation under a scanning electron microscope. An additional portion of the tissue was fixed in neutral buffered formalin, processed, and embedded for histologic sectioning. In all, 5 µm sections were labeled with 4′,6-diamidino-2-phenylindole (DAPI) to demonstrate the absence of nuclei. Additional sections were labeled with antibodies to collagen I (Abcam Ab34710; 1:200), III (1:200), IV (Abcam ab6586; 1:200), and laminin (Abcam AB11575; 1:200) and incubated in AlexaFluor 488 (1:100) secondary antibodies and imaged on an epifluorescent microscope. Remnant DNA size and content were assessed using gel electrophoresis and PicoGreen dsDNA assay. Collagen and sulfated GAG content was quantified through spectrophotometric assays. Additional assessment of nerve-specific growth factor content including NGF, ciliary neurotrophic factor (CNTF), brain-derived neurotrophic factor (BDNF), and NT-3 was performed by ELISA. Quantitative assessments were performed on materials from *n* = 5 individual decellularization procedures and differences between native and decellularized tissues were assessed using a student’s *t* test.

Mechanical properties and gelation temperature of the hydrogels were measured on a dynamic rheometer. The rheometer geometry (40 mm parallel plate) was brought into contact with 1 mL of the sample at a height of 500 µm. The temperature was maintained at 17 °C for 10 minutes, followed by an increase to 37 °C at 2 °C/min, followed by maintenance at 37 °C for 25 mins to demonstrate a 10-minute pre-gelation handling time, assess gelation temperature, and terminal storage and loss moduli. The gelation temperature was the temperature at which the storage modulus begins to rise linearly. The shear storage (G’) and loss moduli (G”) were then measured at 1 Hz and a strain of 5%. Terminal moduli were recorded as the peak values in the plateau region after reaching 37 °C. Assessments were performed on *n* = 3 gels per concentration (10, 20, and 40 mg/mL). The structure of gels at each concentration was then investigated under SEM using the processing methods described above.

### Surgical model and experimental groups

Animal studies were performed in accordance with the guidelines established by the Public Health Service Policy on Humane Care and Use of Laboratory Animals, federal and state regulations, and approved by the Institutional Animal Care and Use Committee (IACUC) at the University of Pittsburgh. Upon entrance into the study, animals were given a 7-day acclimation period prior to any experimental procedures. Record logs of medical procedures were maintained.

Fisher rats were premedicated with subcutaneous buprenorphine (0.1 mg/kg), induced, and maintained using inhaled 2% isoflurane in oxygen at a flow rate of 100–200 mL/min. An incision in the skin was made at the level of the left biceps femoris, the muscle was then dissected along the connective fascia to expose the sciatic nerve. The left sciatic nerve was transected ~5 mm proximal to the bifurcation of the main sciatic branch. An 8 mm nerve segment was then resected proximal to the initial transection to create a gap defect. Gap defects were then repaired using one of five methods: (1–3) 8 mm gap injury repaired with silicone conduits filled with 10, 20, or 40 mg/mL PNM hydrogels (PNM-10, *n* = 8; PNM-20, *n* = 4; and PNM-40, *n* = 4;); (4) 8 mm gap injury repaired with an empty silicone conduit (Conduit, *n* = 8); and (5) 8 mm gap injury repaired with reversed autograft (Autograft, *n* = 8). Uninjured animals (non-injury (NI), *n* = 12) were evaluated as a reference for baseline values.

For the groups receiving a conduit, the nerve stumps were sutured 1 mm into the opposite ends of a 10 mm segment of inert silicone tubing (ID = 2 mm; OD = 3 mm) using two 8–0 Nylon sutures leave an 8 mm gap defect between the two nerve stumps. Different concentrations of PNM or saline were then delivered into the lumen of the conduit with a 25-gauge needle until no remaining air bubbles were observed. The excess solution was removed from the surgical site using a sterile cotton swab. An example of gap creation and PNM deployment is shown in Fig. 1. Autologous grafting was performed by resecting an 8 mm segment similar to gap creation, but the segment was then reversed and sutured back into the gap with two 8–0 Nylon sutures at each end. The muscle and skin were then closed using standard techniques.

### Gait analysis

Nerve functional recovery was assessed longitudinally via motor functional assessment, which was performed using a TSE Systems MotoRater, a semi-automated system for rodent kinematic gait analysis. Animals were evaluated prior to surgery, at weeks 1, 2, 3, 4, every 2 weeks until week 12, and every 4 weeks thereafter until 24 weeks post surgery. During the assessment, rats were placed into a clear square corridor and video of the animal walking was recorded from both lateral planes and from the floor plane of the corridor. Two repeated video recordings were made for each animal at each timepoint. Simi Motion movement analysis software was used to track the animal’s joints throughout the key phases of gait.

Sciatic functional index (SFI) was calculated using standard methods using images captured at mid-stance. Ankle angle was captured at toe-off three measurements per metric were made at each time point for the experimental and contralateral limb per video. A ratio of the average experimental and contralateral limb values was calculated for each video and then the average for the two videos was calculated. Single average values were calculated for each animal at each time point for analysis.

Nerve injuries typically show rapid regeneration acutely until the 10–12 week mark with slower functional gains or plateau in performance thereafter. Therefore, the data were analyzed with a piecewise approach to compare the rate of regeneration within the first half (1–12 weeks) and compare the extent of regeneration in the latter half (12–24 weeks) of the study period. A linear mixed-effects model with time treated continuously was applied to the first 1–12 weeks with post hoc comparisons made between PNM and autograft and PNM and conduit to observe differences in the rate of recovery. Separately, weeks 12, 16, 20, and 24 were compared in a mixed-effects model with time treated categorically to observe differences in the extent of recovery. For comparisons of PNM concentrations, all groups were compared with each other using the same approach.

### Electrophysiology

The electrophysiologic function was assessed by measurement of CMAP at the tibialis anterior (TA) muscle. In brief, prior to euthanasia, animals were premedicated and induced as described above. The sciatic nerve was exposed, a bipolar stimulating probe was placed around the sciatic nerve proximal to the injury site, and two recording electrodes were embedded into the center body of the TA muscle. Stimuli were adjusted via current control from 0.04 mA to 3 mA (intervals of 0.01, 0.1, and 1 mA for each respective order of magnitude), and the maximal response was recorded. At each current, nerves were stimulated 20 times for 200 μs with 400 ms intervals between pulses. The average root means square value of the 20 stimulations at each stimulation amplitude was then calculated using MatLab. A Kruskal–Wallis test with Dunn’s post hoc analysis was used to analyze the data. Post hoc comparisons included PNM vs. Autograft and PNM vs. Conduit.

### Histological evaluation

Animals were killed 24 weeks post surgery following electrophysiologic assessment via an intracardiac KCl injection. Nerves were explanted, subjected to a series of increasing sucrose washes, embedded in optimal cutting temperature medium, and frozen for sectioning. Nerve cross-sections of 10 µm thickness were cut via micro cryotome at the proximal and distal ends of the nerve grafts. Sections were incubated with a neurofilament heavy chain (axons; NF200) antibody (Abcam Ab8135; 1:400) overnight at 4 °C, washed, and incubated again with fluoromyelin red (myelin; Invitrogen F34652; 1:300) and the NF200 secondary antibody (Invitrogen A11008; AlexaFluor 488; 1:1000) for 1 h at room temperature in the dark. Sections were then coverslipped in an aqueous mounting media containing DAPI. The number of axons was quantified by using images taken at ×4 magnification (to capture the entire nerve cross-section in a single image) using a counting algorithm in Cell Profiler Software. Among groups, the distal location was selected for comparison using a Kruskal–Wallis test with Dunn’s post hoc analysis comparing PNM vs. Autograft and PNM vs. Conduit. All groups within each group, proximal and distal axon counts were compared using a Wilcoxon matched-pairs signed-rank test. For comparisons of PNM concentrations, all groups were compared with each other using the same approach.

### Reporting summary

Further information on research design is available in the Nature Research Reporting Summary linked to this article.

## Supplementary information

Reporting Summary

Supplementary Figure 1

## Data Availability

The data sets generated during and/or analyzed during the current study are available from the corresponding author on reasonable request.
