# Supplementary material for: Nerve-specific extracellular matrix hydrogel promotes functional regeneration following nerve gap injury
Source: NPJ Regen Med. 2021 Oct 25;6:69. doi: 10.1038/s41536-021-00174-8 (PMC8546053; doi:10.1038/s41536-021-00174-8)
Supplement: Supplementary file 1 — Reporting Summary [file 41536_2021_174_MOESM1_ESM.pdf]

nature research

Corresponding author(s): Bryan Brown

Last updated by author(s): May 13, 2021

Reporting Summary

Nature Research wishes to improve the reproducibility of the work that we publish. This form provides structure for consistency and transparency in reporting. For further information on Nature Research policies, see our Editorial Policies and the Editorial Policy Checklist.

Statistics

For all statistical analyses, confirm that the following items are present in the figure legend, table legend, main text, or Methods section.

n/a	Confirmed	
☐	☒	The exact sample size (*n*) for each experimental group/condition, given as a discrete number and unit of measurement
☐	☒	A statement on whether measurements were taken from distinct samples or whether the same sample was measured repeatedly
☐	☒	The statistical test(s) used AND whether they are one- or two-sided<br>*Only common tests should be described solely by name; describe more complex techniques in the Methods section.*
☐	☒	A description of all covariates tested
☐	☒	A description of any assumptions or corrections, such as tests of normality and adjustment for multiple comparisons
☐	☒	A full description of the statistical parameters including central tendency (e.g. means) or other basic estimates (e.g. regression coefficient) AND variation (e.g. standard deviation) or associated estimates of uncertainty (e.g. confidence intervals)
☐	☒	For null hypothesis testing, the test statistic (e.g. *F*, *t*, *r*) with confidence intervals, effect sizes, degrees of freedom and *P* value noted<br>*Give P values as exact values whenever suitable.*
☒	☐	For Bayesian analysis, information on the choice of priors and Markov chain Monte Carlo settings
☒	☐	For hierarchical and complex designs, identification of the appropriate level for tests and full reporting of outcomes
☒	☐	Estimates of effect sizes (e.g. Cohen's *d*, Pearson's *r*), indicating how they were calculated

Our web collection on statistics for biologists contains articles on many of the points above.

Software and code

Policy information about availability of computer code

Data collection	Matlab was used to collect electrophysiology data recordings and to determine root mean square values. SimiMotion software was used to analyze gait parameters. ImageJ was used to quantify axon populations.
Data analysis	Statistical analyses were performed in GraphPad PRISM. Microsoft Excel and GraphPad PRISM were used to prepare graphs presented in the manuscript. Linear mixed models were performed in SPSS by IBM.

For manuscripts utilizing custom algorithms or software that are central to the research but not yet described in published literature, software must be made available to editors and reviewers. We strongly encourage code deposition in a community repository (e.g. GitHub). See the Nature Research guidelines for submitting code & software for further information.

Data

Policy information about availability of data

All manuscripts must include a data availability statement. This statement should provide the following information, where applicable:

- Accession codes, unique identifiers, or web links for publicly available datasets
- A list of figures that have associated raw data
- A description of any restrictions on data availability

The datasets generated during and/or analysed during the current study are available from the corresponding author on reasonable request.

Field-specific reporting

Please select the one below that is the best fit for your research. If you are not sure, read the appropriate sections before making your selection.

☒ Life sciences ☐ Behavioural & social sciences ☐ Ecological, evolutionary & environmental sciences

For a reference copy of the document with all sections, see nature.com/documents/nr-reporting-summary-flat.pdf

Life sciences study design

All studies must disclose on these points even when the disclosure is negative.

Sample size	Sample sizes were determined based upon previous experience. A power analysis was conducted to initially estimate the total number of animals needed to observe a significant difference between groups.
Data exclusions	No data were excluded from this study.
Replication	No attempts have been made to replicate the findings of the present study to-date. However, all results are in line with those previously reported for the use of this material in nerve repair, as is described in the discussion of the manuscript. For in vitro testing and characterization of the decellularized nerve materials, 5 individual decellularization batches were tested, representing biological replicates, not technical replicates.
Randomization	Animals were randomly allocated to surgical groups at the time of surgery.
Blinding	Blinding of the surgeon was not possible due to the distinct nature of each individual surgical procedure. Electrophysiologic and gait testing were performed by individuals blinded to animal group. Computerized gait analysis and histologic assessments were not blinded.

Reporting for specific materials, systems and methods

We require information from authors about some types of materials, experimental systems and methods used in many studies. Here, indicate whether each material, system or method listed is relevant to your study. If you are not sure if a list item applies to your research, read the appropriate section before selecting a response.

Materials & experimental systems

n/a	Involved in the study	
☐	☒	Antibodies
☒	☐	Eukaryotic cell lines
☒	☐	Palaeontology and archaeology
☐	☒	Animals and other organisms
☒	☐	Human research participants
☒	☐	Clinical data
☒	☐	Dual use research of concern

Methods

n/a	Involved in the study	
☒	☐	ChIP-seq
☒	☐	Flow cytometry
☒	☐	MRI-based neuroimaging

Antibodies

Antibodies used	Primary antibodies:<br>Collagen I: (1:200) C2456 by Sigma<br>Collagen III: (1:200) C7805 by Sigma<br>Collagen IV: (1:200) C1926 by Sigma<br>Laminin: ab11575 (1:200) by Abcam<br>NF200: ab8135 (1:400) by Abcam<br>Fluoromyelin: F34652 (1:300) by Invitrogen (not an antibody, requires no secondary)
Validation	All antibodies used were validated by the manufacturers as listed below:<br>Collagen I: C2456 reacts with: bovine, human, rabbit, deer, rat, pig. Suitable for: dot blot, immunohistochemistry, ELISA<br>Collagen III: C7805 reacts with: human, rat. Suitable for: dot blot, immunohistochemistry, ELISA, western blot<br>Collagen IV: C1926 reacts with: human. Suitable for: dot blot, immunohistochemistry, ELISA, microarray<br>Laminin: ab11575 Reacts with: Mouse, Human. Predicted to work with: Rat, Horse, Dog, Pig, Xenopus laevis, Reptile, Mammals, Amphibian. Suitable for: Dot blot, IHC-P<br>NF200: ab8135 reacts with: Mouse, Rat. Suitable for: IHC, WB<br>Fluoromyelin: F34652 reacts with any myelin structures, not an antibody and suitable for any application containing intact myelin.

Animals and other organisms

Policy information about studies involving animals; ARRIVE guidelines recommended for reporting animal research

Laboratory animals	Equivalent numbers of 8-12 week old Fisher rats (Charles River) were used in this study.
Wild animals	This study did not involve wild animals
Field-collected samples	This study did not involve field-collected samples
Ethics oversight	The experimental design and rationale for animal use were reviewed and approved by the University of Pittsburgh Animal Care and Use Committee.

Note that full information on the approval of the study protocol must also be provided in the manuscript.
