# Supplementary figures and images for: Nerve-specific extracellular matrix hydrogel promotes functional regeneration following nerve gap injury
Source: NPJ Regen Med. 2021 Oct 25;6:69. doi: 10.1038/s41536-021-00174-8 (PMC8546053; doi:10.1038/s41536-021-00174-8)

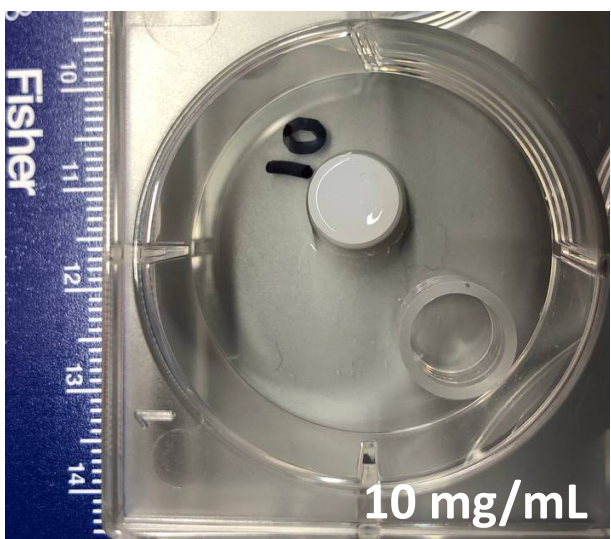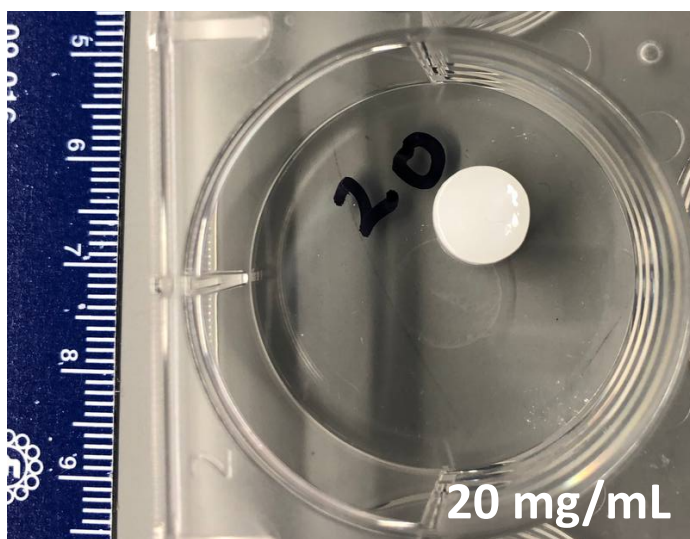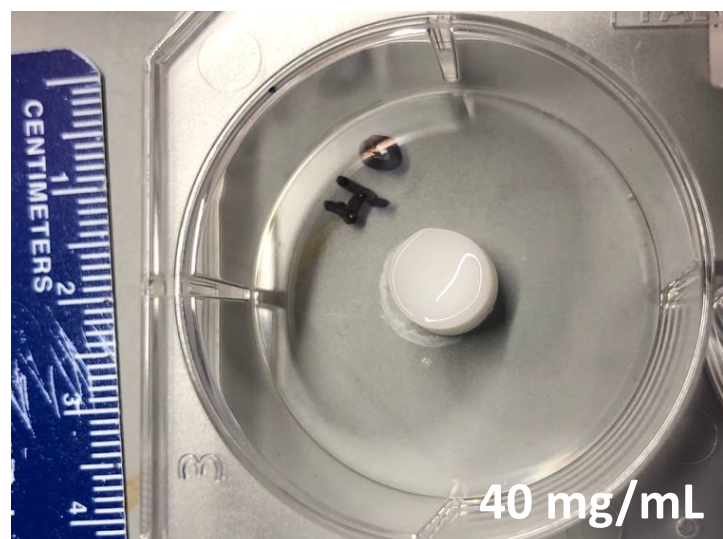

Supplement: Supplementary file 2 — Supplementary Figure 1 [file 41536_2021_174_MOESM2_ESM.pdf]
